# A thermodynamic framework to identify apposite refrigerant former for hydrate-based applications

**DOI:** 10.1038/s41598-022-19557-y

**Published:** 2022-10-06

**Authors:** Harshal J. Dongre, Aman Deshmukh, Amiya K. Jana

**Affiliations:** Energy and Process Engineering Laboratory, Department of Chemical Engineering, Indian Institute of Technology Kharagpur, Kharagpur, 721302 India

**Keywords:** Energy science and technology, Engineering, Mathematics and computing

## Abstract

High latent heat storage capacity with naturally assisted salt rejection makes the clathrate compounds appropriate for applications towards load management and desalination processes. Adding to these energy savings are the ease of operations provided by water and the mild conditions at which the refrigerant hydrates are occurred. A direct comparison between these hydrates becomes unfeasible due to the scattered experimental data. Though thermodynamics can streamline this dispersed data, they are currently limited to being a proof of concept most accurately representing the experimental observations. We address this critical deficit of phase assessment and identify, from among R13, R14, R22, R23, R125, R134a and R152a, the most suitable hydrate former for the concerned application. An approach based on van der Waals and Platteeuw model is undertaken and the estimates are quantified in terms of percent average absolute relative deviations (% AARD). An average AARD of 1.75% and 2.68% is observed in pure and aqueous electrolytic phase of NaCl, KCl, CaCl_2_ and MgCl_2_, respectively. The model predictions are then estimated at temperature/salinity of 281 K/0 wt% and 284 K/3.5 wt%. Together with the qualitative assessment of the hydrate phase, viz, vapor pressure, compressibility and dissociation enthalpy, R152a refrigerant is observed to be the appropriate former for applications to both load management and desalination.

## Introduction

The efficient management of the available resources is necessary to sustainably meet the rising energy demands. This rise is predominantly fuelled by a desire for comfortable living and working space, requiring the principally regulatory space conditioning systems^1^. Since this regulatory behavior is load and time-dependent, a large amount of energy is wasted during downtimes. A distributed load management system (DLMS) aims to flatten these peaks in energy demands, facilitating efficient usage of available resources^2^. To store and exchange heat with the space conditioning systems, the DLMS employs phase change material (PCM) like eutectic salts, which exhibit characteristics like the high density of fusion (heat) and thermal conductivity. However, operations involving eutectic salts as PCM necessitate a high degree of subcooling. This increased operating cost, combined with the corrosive tendency of salts toward equipment, result in uncertainty towards their prolonged usage^3^. Gas hydrates exhibit properties similar to eutectic salts; fine-tuning further by additives like salts, alcohols and nanoparticles makes them suitable as PCM^4^. Concurrently, as water freezes, the salt ions are impartially rejected from hydrate crystals. These crystals on melting yield freshwater, thereby achieving seawater desalination^5^. This hydrate-based desalination (HBD) process is not susceptible to the chlorine and temperature accelerated corrosion, which accounts for the bulk of operating and maintenance costs of membrane and distillation technologies, respectively^6^.

The gas hydrates are inclusion compounds of water molecules structurally held together by hydrogen bonding. Guest molecules like lower hydrocarbons, sour gases and refrigerants enter these cages, imparting stabilizing energy to the structure^7^. The occupancy in these cages is governed by non-polarity and size of the guest molecule. The extent of interactions between the constituting molecules of the hydrate lattice then characterizes the stabilizing pressure. The conducive low temperature and high pressure of the ocean floor result in natural deposits of methane hydrates^7^. However, their commercial application towards refrigeration and cold storage, desalination, and gas fractionation and storage are primarily dependent on the guest properties. Studies on pure hydrates of methane^8^ or carbon dioxide^9^ observe a high equilibrium pressure requirement. However, the accidental release of these greenhouse gases from the dense hydrates can negatively impact the environment. The use of thermodynamic promoters like tetrahydrofuran (THF)^10^, cyclopentane (CP)^11,12^, and cyclohexane (CH)^13^ help to reduce the requirement of high equilibrium hydrate formation pressure. However, added promoter increases the degree of subcooling needed for hydrate nucleation, resulting in unwanted ice crystals^14^. The consequent changes to rheological properties affect the fluidity, thereby impacting the heat transfer rate for the DLMS cyclic processes^15^. When used for desalination, the separation of promoter from hydrate emulsion requires additional equipment, increasing the total cost of HBD^16^. On the other hand, pure hydrates of water soluble formers like THF, TBAB and TBPB are occurred at lower equilibrium pressures^17^. However, it comes at the expense of low energy density and non-uniform hydrate nucleation, increasing the size and cost of processes equipment.

The hydrates of refrigerant formers distinctly offer properties conducive to hydrate applications, including high energy density^18^, good thermal conductivity^4^, and mild formation temperature and pressure conditions^19^. The inhibitory nature of salt towards hydrate formation results in much higher equilibrium pressure, increasing the operating costs. Since pure refrigerant hydrates occur at lower pressures, the inhibition effect of salt does not significantly increase the equilibrium pressures. In this regard, the applicability of refrigerant hydrates towards refrigeration and cold storage is reported for R11^2^, R12^2^, R32^20^, R125^20^, R134a^21^ and their mixtures^22^. Moreover, the heat transfer and flow properties of the hydrate slurry can be modified with additives like glycols, NaCl, and aluminium, copper and graphene nanoparticles^4^. To circumvent the high-pressure requirement for an economic HBD process, preliminary studies employing propane and R12 report a salt removal efficiency of 31% from a 2.5 wt% NaCl solution^23^. As a hydrate former for HBD, the use of cyclopentane leads to a removal efficiency between 60 and 96% using distinct crystal processing technologies^5,16^. However, the additional cost associated with the separation of the hydrate former from the emulsion obtained from dissociated hydrate crystals cannot be overlooked. Even when used as a pre-treatment to the conventional reverse osmosis desalination process, the water flux is reported to increase by over 28 times the raw seawater^24^. The lack of common conditions makes a comparison between the different experimental studies difficult. Advantageously, the coexistence of hydrate with the aqueous phase, when expressed thermodynamically, can help identify the best suited former.

Numerous studies employ the definitive van der Waals and Platteeuw^25^ (vdWP) model to describe the hydrate phase equilibrium conditions for pure and mixed guests^26^. This model is based on the equality of chemical potential of water between the equilibrium phases, viz., ice, bulk water, and hydrate. A statistical interpretation of Langmuir adsorption theory is then used to describe water's chemical potential in the hydrate, while for the bulk aqueous phase, Holder's equation^27^ is used. The available literature interprets the equilibrium vapor phase using the various equation of state models like Soave–Redlich–Kwong (SRK)^28,29^, Cubic-Plus-Association (CPA)^22,29^, Valderrama–Patel–Teja (VPT)^29^, simplified Perturbed-Chain Statistical Associating Fluid Theory (sPC-SAFT)^30^, Peng–Robinson–Stryjek–Vera (PRSV)^18^, Peng Robinson (PR)^31^, or the ideal gas law ^32^. Concurrently, the nonideality of the equilibrium bulk aqueous phase due to the presence of refrigerant is modelled using the UNIFAC activity coefficient model^18^ or ignoring this influence ^13,28,30,32^. However, the electrolyte has a significant effect on the activity of water and is described using the Aasberg–Petersen approach^31,33^, the Debye Huckel theory^22^, or the Pitzer model^28,34^. Commonly, all these different approaches employ the parameters of Kihara potential for regression to the experimental data. The Kihara spherical cell function accounts for the intermolecular potential between the guest and a water molecule constituting the cavity. The Langmuir adsorption constant is then approximated based on guest interactions with all molecules of the host cavity.

The gas hydrates of refrigerant formers can potentially serve towards distributed load management system and hydrate based desalination. The lack of uniform experimental phase equilibrium data impedes a detailed comparison. The thermodynamic model can help discriminate between these refrigerant formers, but they currently serve to accurately represent the experimental data and lack in the use of qualitative assessments they provide. To address this research gap, we propose a simplified thermodynamic phase equilibrium model based on the van der Waals and Platteeuw^25^ description of the hydrate phase. The equilibrium vapor phase is modelled using the Patel Teja^35^ equation of state (EoS), while the non-ideality of water due to the presence of guests and salt is described using the UNIQUAC^36^ and Pitzer^37^ activity coefficient model, respectively. The model estimates are compared to those available in the literature. The model predictions together with the hydrate phase properties are used to identify the best suited former for each application.

## Thermodynamic modelling of phase equilibria

Hydrate phase equilibrium model formulates the interaction between the coexisting phases viz., hydrate, bulk water (or ice) and vapor. In this light, we propose to employ the vdWP theory, which describes these phase interactions in terms of alteration to the chemical potential of water due to hydrate formers^25^. This theory assumes that a guest molecule gets adsorbed into a hypothetical empty hydrate lattice, thereby stabilizing it. This adsorption is further described by the Langmuir adsorption theory, resulting in fractional occupancy of the lattice cages. This occupancy is governed by the extent of interaction between the guest and host molecules. These host molecules either take the form of dodecahedra (5^12^), tetrakaidecahedron (5^12^6^2^) or hexakaidecahedron (5^12^6^4^). Two small (5^12^) and six large (5^12^6^2^) voids assemble to form the unit lattice of cubic sI structure. While, that of sII structure contains sixteen small (5^12^) and eight large (5^12^6^4^) voids. The intermolecular interaction is then described by the Kihara spherical shell potential. The equilibrium vapor and liquid phases are described using the Patel Teja EoS^35^ and UNIQUAC activity coefficient model^36^, respectively. The nonideality of water due to electrolyte addition is modelled by Pitzer activity coefficient model^37^.

### Hydrate phase model

The statistical thermodynamic model of van der Waals and Platteeuw^25^ describes the change in chemical potential of water $\left( {\mu_{{\text{w}}} } \right)$ due to occupying a hypothetical empty hydrate lattice by hydrate former as,

1 $$ \Delta \mu_{{\text{w}}}^{{\beta - {\text{H}}}} = - RT \, \sum\limits_{m} \nu_{m} {\text{ln}}\left( {1 - \sum\limits_{j} \, \theta_{mj} } \right) $$

here, $R$ is the universal gas constant, $T$ the temperature, $\nu_{m}$ the coordination number indicating the number of water molecules per $m$ type cavity and $\theta_{mj}$ the fraction of cages occupied by guest $j$. Furthermore, the fractional occupancy of each cavity type is estimated based on the Langmuir adsorption theory as,

2 $$ \theta_{mj} = \frac{{C_{mj} f_{j} }}{{1 + \sum\limits_{m} \, C_{mj} f_{j} }} $$

where, $C_{mj}$ represents the Langmuir constant, estimated as,

3 $$ C_{mj} = \frac{4\pi }{{kT}} \, \int_{0}^{{R_{{{\text{cell}}}} - a}} {\text{ exp}}\left( {\frac{ - W(r)}{{kT}}r^{2} } \right){\text{dr}} $$

here, $k$ is the Boltzmann constant and $W(r)$ the cell potential function accounting the guest interactions with the host cavity. This cell potential function is estimated as,

4 $$ W(r) = 2z\varepsilon \left[ {\frac{{\sigma^{12} }}{{R_{{{\text{cell}}}}^{11} r}}\left( {\delta^{10} + \frac{a}{r}\delta^{11} } \right) - \frac{{\sigma^{6} }}{{R_{{{\text{cell}}}}^{5} r}}\left( {\delta^{4} + \frac{a}{r}\delta^{5} } \right)} \right] $$

where, $z$ is the coordination number, $\varepsilon$ the depth of potential well, $\sigma$ the collision diameter, $R_{cell}$ the radius of cell cavity, $a$ the core radius and

5 $$ \delta = {{\left[ {\left( {1 - \frac{r}{{R_{{{\text{cell}}}} }} - \frac{a}{{R_{{{\text{cell}}}} }}} \right)^{ - N} - \left( {1 + \frac{r}{{R_{{{\text{cell}}}} }} - \frac{a}{{R_{{{\text{cell}}}} }}} \right)^{ - N} } \right]} \mathord{\left/ {\vphantom {{\left[ {\left( {1 - \frac{r}{{R_{{{\text{cell}}}} }} - \frac{a}{{R_{{{\text{cell}}}} }}} \right)^{ - N} - \left( {1 + \frac{r}{{R_{{{\text{cell}}}} }} - \frac{a}{{R_{{{\text{cell}}}} }}} \right)^{ - N} } \right]} N}} \right. \kern-\nulldelimiterspace} N} $$

The hydrate structure crystal data are tabulated in Table 1. Moreover, in Eq. (2), the term $f_{j}$ represents the fugacity of guest which is estimated using the Patel Teja EoS^35^.

**Table 1:** Structural crystallographic properties of different hydrate structure and their reference parameters.

Structure	Cage type	Cell radius, *R*_cell_ (Å)	Coordination number, *z*	$\Delta \mu_{{\text{w}}}^{{\beta - {\text{L}},{\text{o}}}}$	$\Delta h_{{\text{w}}}^{{\text{o}}}$		$\Delta V_{{\text{w}}}^{{\text{o}}}$	
Phase					Ice	Water	Ice	Water
Si	5^12^	3.95	20	1297	1151	−4858	3	4.6
	5^12^6^2^	4.33	24					
sII	5^12^	3.91	20	883	808	−5201	3.4	5
	5^12^6^4^	4.73	28					

At equilibrium, the change in the chemical potential of water in the hydrate phase is compensated by a correspondingly similar change in the bulk phase when the hypothetical lattice forms. This change in the chemical potential of water is estimated using the simplified form, proposed by Holder’s equation^27^ as,

6 $$ \frac{{\Delta \mu_{{\text{w}}}^{{\beta - {\text{aq}}}} }}{RT} = \frac{{\Delta \mu_{{\text{w}}}^{{\beta - {\text{L}},{\text{o}}}} }}{{RT_{{\text{o}}} }} - \int\limits_{{T_{{\text{o}}} }}^{T} {\frac{{\Delta h_{{\text{w}}} }}{{RT^{2} }}} {\text{ d}}T + \int\limits_{{P_{{\text{o}}} }}^{P} {\frac{{\Delta V_{{\text{w}}} }}{RT} \, } {\text{d}}P - \ln \left( {\gamma_{{\text{w}}} x_{{\text{w}}} } \right) $$

Here, the term $\Delta \mu_{{\text{w}}}^{{\beta - {\text{L,o}}}}$ in the above Eq. (6), represents the chemical potential difference estimated at a reference temperature and pressure of 273.15 K and 0 Pa, respectively. Furthermore, the symbols, $\Delta h_{{\text{w}}}$ and $\Delta V_{{\text{w}}}$, describe respectively the molar differences in specific heat and volume of water between the hydrate and bulk water phase. These are estimated as,

7 $$ \Delta h_{{\text{w}}} = \Delta h_{{\text{w}}}^{{\text{o}}} + \int\limits_{{T_{{\text{o}}} }}^{T} {\left[ { - 38.12 + 0.141(T - T_{{\text{o}}} )} \right]dT} $$

8 $$ \Delta V_{{\text{w}}} = \Delta V_{{\text{w}}}^{{\text{o}}} + 6.695 \times 10^{ - 15} P $$

The reference molar properties of enthalpy ($\Delta h_{{\text{w}}}^{{\text{o}}}$) and volume ($\Delta V_{{\text{w}}}^{{\text{o}}}$) are evaluated at 273.15 K and 0 Pa. The reference properties used in the equilibrium modelling are charted in Table 1. Finally, in Eq. (6), $\gamma_{{\text{w}}}$ denotes the activity coefficient of water and $x_{{\text{w}}}$ the mole fraction of water in liquid phase.

### Liquid phase

The extent of intermolecular interactions is quantified in terms of activity and is much influenced by electrolytes than non-electrolytes. Thus, to account for the influence of ions and hydrate formers on the ideal behaviour of water, Pitzer^37^ and UNIQUAC^36^ activity coefficient models are used, respectively. The combined effect on the water is therefore, expressed by a $\gamma + \gamma$ approach as,

9 $$ \ln \gamma = \ln \gamma_{{\text{s}}} + \ln \gamma_{{\text{g}}} $$

where, $\gamma$ indicates the activity coefficient of water while subscripts s and g depict salt and guest species, respectively. Pitzer model^37^ expresses the coefficient $\gamma_{{\text{s}}}$ in terms of influence of salt ions on the osmotic coefficient, $\phi$ of water as,

10 $$ \ln (\gamma_{{\text{w}}} x_{{\text{w}}} ) = - \frac{MW}{{1000}}\left( {\sum\limits_{i = 1}^{n} {m_{i} } } \right)\phi $$

where, $MW$ is the molecular weight of water, $m_{i}$ the molality of concerned ions and the osmotic coefficient is,

11 $$ \left( {\sum {m_{i} } } \right)\left( {\phi - 1} \right) = 2\left\{ \begin{gathered} \frac{{A^{\phi } I^{1.5} }}{{1 + 1.2I^{0.5} }} + \sum\limits_{c} {\sum\limits_{a} {m_{c} m_{a} (B_{ca}^{\phi } + ZC_{ca} )} } \hfill \\ \sum {\sum\limits_{c < c^{\prime}} {m_{c} m_{c^{\prime}} (\phi_{{cc^{^{\prime}} }}^{\varphi } + \sum\limits_{a} {m_{a} \psi_{cc^{\prime}a} } )} } + \sum {\sum\limits_{a < a^{\prime}} {m_{a} m_{a^{\prime}} (\phi_{aa^{\prime}} + \sum\limits_{c} {m_{a} \psi_{aa^{\prime}c} } )} } \hfill \\ + \sum\limits_{n} {\sum\limits_{c} {m_{n} m_{c} \lambda_{nc} } } + \sum\limits_{n} {\sum\limits_{a} {m_{n} m_{a} \lambda_{na} } } + \sum\limits_{n} {\sum\limits_{c} {\sum\limits_{a} {m_{n} m_{c} m_{a} \zeta_{nca} } } } \hfill \\ \end{gathered} \right\} $$

here, $I$ is the ionic strength of the solution, while $\lambda$ and $\zeta$ denote the binary and ternary ionic interaction between cation, anion and neutral species which are indicated by subscripts $c$, $a$ and $n$, respectively. These ionic interaction parameters are obtained by regressing the above equation to the experimental hydrate phase equilibrium data of aqueous salt solutions. Furthermore, in Eq. (11),

12 $$ I = \frac{1}{2}\sum\limits_{l = c,a} {m_{l} z_{l}^{2} } $$

13 $$ Z = \sum\limits_{l = c,n} {m_{l} \left| {z_{l} } \right|} $$

while,

14 $$ C_{ca} = \frac{{C_{ca}^{\phi } }}{{2\sqrt {\left| {z_{c} z_{a} } \right|} }} $$

15 $$ B_{ca}^{\phi } = \beta_{ca}^{(0)} + \beta_{ca}^{\left( 1 \right)} e^{{ - \alpha_{ca} \sqrt I }} + \beta_{ca}^{2} e^{ - 12\sqrt I } $$

16 $$ \phi_{mn}^{\phi } = \theta_{mn} + {}^{E}\theta_{mn} \left( I \right) + I^{E} \theta_{mn}^{^{\prime}} \left( I \right) $$

The terms $C_{ca}$, $B_{ca}^{\phi }$ and $\phi_{mn}^{\phi }$ are temperature dependent coefficients of the Pitzer model and values for these are adopted from Spencer et al.^38^ Here, $\alpha_{ca}$ is adopted as 2 and 1.4 for univalent and higher valence ions, respectively^37^.

On the other hand, the nonideality added to water properties due to solvation of hydrate former is modelled using the UNIQUAC activity coefficient model^36^. This model describes the total molar excess Gibbs free energy ($G^{{\text{E}}}$), expressed as a sum of combinatorial ($G^{{\text{C}}}$) and residual energies ($G^{{\text{R}}}$), which is further correlated in terms of activity coefficient of species, $i$ as,

17 $$ \ln \left( {\gamma_{i} } \right) = \ln \left( {\gamma_{i}^{{\text{C}}} } \right) + \ln \left( {\gamma_{i}^{{\text{R}}} } \right) $$

Of these, the combinatorial part ($\gamma_{i}^{{\text{C}}}$) is expressed as,

18 $$ \ln \left( {\gamma_{i}^{{\text{C}}} } \right) = \ln \left( {\frac{{\phi_{i} }}{{x{}_{i}}}} \right) + \frac{Z}{2}q_{i} \ln \left( {\frac{{\theta_{i} }}{{x_{i} }}} \right) + l_{i} - \left( {\frac{{\phi_{i} }}{{x_{i} }}} \right)\sum\limits_{j = 1}^{N} {x_{j} l_{j} } $$

while, the residual part ($\gamma_{i}^{{\text{R}}}$) is,

19 $$ \ln \gamma_{i}^{R} = q_{i} \left[ {1 - \ln \left( {\sum\limits_{j = 1}^{n} {\theta_{j} \tau_{ji} } } \right) - \sum\limits_{j = 1}^{n} {\left( {\frac{{\theta_{j} \tau_{ij} }}{{\sum\limits_{k = 1}^{n} {\theta_{k} \tau_{kj} } }}} \right)} } \right] $$

In the Eq. (18), $\phi_{i}$ and $\theta_{i}$ are the effective volume and area fraction, respectively, $j$ the dummy index expressing all the components in the system and the parameter $l_{i}$ is,

20 $$ l_{i} = \frac{Z}{2}(r_{i} - q_{i} ) - r_{i} + 1 $$

where, $Z$ is the coordination number equalling 10 while $r_{i}$ and $q_{i}$ are volume and surface parameter, respectively. Furthermore, the effective volume and area fractions are estimated as,

21 $$ \theta_{i} = \frac{{x_{i} q_{i} }}{{\sum\limits_{j = 1}^{N} {x_{j} } q_{j} }} $$

and

22 $$ \phi_{i} = \frac{{x_{i} r_{i} }}{{\sum\limits_{j = 1}^{n} {x_{i} r_{j} } }} $$

In the residual contributions of Eq. (19), the term $\tau$ is the binary interaction parameters between species $i$ and $k$, such that $\tau_{ki} \ne \tau_{ik}$ and is given as,

23 $$ \tau_{ki} = \exp \left[ { - \frac{{u_{ki} - u_{ii} }}{RT}} \right] $$

Finally, to estimate the mole fraction of water ($x_{{\text{w}}}$) required in evaluation of chemical potential of the bulk phase, its composition and hydrate former solubility are used. The mole fraction of guest $i$ in the liquid phase is estimated using the Krichevsky–Kasarnovski equation^39^,

24 $$ f_{i}^{{\text{L}}} = x_{i} H_{{i{\text{w}}}} \exp \left( {\frac{{\overline{v}_{i}^{\infty } (P - P_{{\text{w}}}^{{{\text{sat}}}} )}}{RT}} \right) $$

where, $\overline{v}_{i}^{\infty }$ is the infinite partial molar volume, $P_{{\text{w}}}^{{{\text{sat}}}}$ the vapor pressure of water and $H_{{i{\text{w}}}}$ the Henry’s constant of the gas component, estimated using a temperature dependent form as,

25 $$ - \ln H_{i} = \frac{1}{R}\left( {H_{i,1} + \frac{{H_{i,2} }}{T} + H_{i,3} \ln (T) + H_{1,4} T} \right) $$

The individual parameters of Eq. (25) are tabulated in Table 2.

**Table 2:** Ranges of phase equilibrium data for refrigerant hydrates in different aqueous media.

Hydrate former	Salt	Temperature range (K)	Pressure range (MPa)	Salt range (mol/kg)	References
R13 (CFC)	–	273.25–281.15	0.324–2.140	–	^40^
	NaCl	271.65–279.65	0.303–2.030	0.427	^40^
R14 (PFC)	–	273.49–281.19	3.400–12.90	–	^41^
R22 (CHFC)	–	274.90–285.90	0.113–0.506	–	^42^
	–	277.80–289.40	0.154–0.773	–	^43^
	–	267.65–289.45	0.066–0.773	–	^44^
	–	276.30–290.10	0.141–0.830	–	^45^
	NaCl	274.00–287.30	0.167–0.775	0.856–2.568	^45^
	KCl	275.10–287.80	0.140–0.790	0.670–2.010	^45^
	MgCl_2_	274.00–287.30	0.167–0.775	0.525–1.575	^45^
R23 (HFC)	–	275.40–285.80	0.450–1.566	–	^42^
	–	282.52–292.28	0.920–3.850	–	^41^
	–	273.55–291.75	0.353–3.650	–	^40^
	–	275.40–292.00	0.450–3.440	–	^46^
	NaCl	270.35–292.55	0.305–3.440	0.341–0.854	^40^
R134a (HFC)	–	274.40–282.20	0.065–0.345	–	^42^
	–	274.75–283.15	0.072–0.414	–	^47^
	–	265.31–273.51	0.039–0.414	–	^48^
	–	274.04–283.42	0.055–0.409	–	^49^
	–	275.00–283.17	0.062–0.412	–	^50^
	NaCl	268.10–280.60	0.086–0.383	0.090–3.020	^33^
	MgCl_2_	274.70–282.30	0.116–0.410	0.259–0.868	^31^
	CaCl_2_	276.20–281.20	0.125–0.392	0.358–0.756	^51^
R152a (HFC)	–	264.72–288.15	0.049–0.443	–	^48^
	–	273.65–287.35	0.065–0.399	–	^40^
	NaCl	270.95–286.65	0.015–0.413	0.438–0.899	^40^
R32 (HFC)	–	274.42–292.45	0.190–1.340	–	^49^
	–	275.50–292.65	0.200–1.370	–	^50^
R125 (HFC)	–	274.60–284.30	0.117–1.030	–	^42^
	–	274.50–283.64	0.126–0.854	–	^49^
	–	274.94–283.70	0.117–0.870	–	^50^
R410a (HFC)	–	277.00–292.50	0.178–1.365	–	^42^
	–	278.28–292.22	0.257–1.324	–	^49^
	–	277.50–293.00	0.179–1.421	–	^22^
CH_4_ (HC)	–	273.70–315.1	2.770–237.5	–	^7^
C_2_H_6_ (HC)	–	273.70–287.40	0.510–3.298	–	^7^
C_3_H_8_ (HC)	–	273.20–278.40	0.165–0.542	–	^7^
CO_2_	–	273.36–283.30	1.388–4.468	–	^7^

### Vapor phase

To correlate the vapor and liquid phases at equilibrium, Patel Teja EoS^35^ is employed here which can be expressed as,

26 $$ P = \frac{RT}{{v - b}} - \frac{a}{v(v + b) + c(v - b)} $$

where,

27 $$ a = \frac{{\Omega_{{\text{a}}} \alpha \left( {T_{R} } \right)R^{2} T_{c}^{2} }}{{P_{c} }} $$

28 $$ b = \frac{{\Omega_{{\text{b}}} RT_{c} }}{{P_{c} }} $$

29 $$ c = \frac{{\Omega_{{\text{c}}} RT_{c} }}{{P_{c} }} $$

here, $T_{c}$, $P_{c}$ and $T_{R}$ are the critical temperature, critical pressure and reduced temperature $\left( {T/T_{c} } \right)$, respectively. The parameter, $\alpha \left( {T_{R} } \right)$ is estimated as,

30 $$ \alpha \left( {T_{r} } \right){\text{) = exp}}\left[ {H_{1} \left( {1 - \frac{T}{{T_{c} }}} \right)^{{H_{2} }} } \right] $$

Further, in equations. (27)-(29), the parameters $\Omega_{{\text{a}}}$ and $\Omega_{{\text{c}}}$ are evaluated as,

31 $$ \Omega_{{\text{a}}} = 3\zeta_{{\text{c}}}^{2} + 3(1 - 2\zeta_{{\text{c}}} )\Omega_{{\text{b}}} + \Omega_{{\text{b}}}^{2} + 1 - 3\zeta_{{\text{c}}} $$

32 $$ \Omega_{{\text{c}}} = 1 - 3\zeta_{{\text{c}}} $$

where, $\Omega_{{\text{b}}}$ is the smallest positive real root of,

33 $$ \Omega_{{\text{b}}}^{3} + 3\zeta_{{\text{c}}}^{2} \Omega_{{\text{b}}} + (2 - 3\zeta_{{\text{c}}} )\Omega_{{\text{b}}}^{2} - \zeta_{{\text{c}}}^{3} = 0 $$

and $\zeta_{{\text{c}}}$ is correlated in terms of the acentric factor, $\omega$ as,

34 $$ \zeta_{{\text{c}}} = 0.3272 - 0.0537\omega - 0.0147\omega^{2} ; $$

Expressing the cubic PT-EoS in terms of compressibility factor $\left( Z \right)$,

35 $$ Z^{3} + (C - 1)Z^{2} + (A - 2BC - B^{2} - B - C)Z + (BC + C - A)B = 0 $$

where, *A, B* and* C* are evaluated as,

36 $$ A = \frac{aP}{{R^{2} T^{2} }} $$

37 $$ B = \frac{bP}{{RT}} $$

38 $$ C = \frac{cP}{{RT}} $$

For a pure component system, the fugacity coefficient is estimated as,

39 $$ \ln \left( \phi \right) = Z - 1 - \ln \left( {Z - B} \right) + \frac{a}{2RTN}\ln \left( {\frac{Z + M}{{Z + Q}}} \right) $$

where, parameters *M, N* and *Q*

40 $$ M = \left( {\frac{b + c}{2} - N} \right)\frac{P}{RT} $$

41 $$ N = \left( {bc + \frac{{\left( {b + c} \right)^{2} }}{2}} \right)^{ - 1/2} $$

42 $$ Q = \left( {\frac{b + c}{2} + N} \right)\frac{P}{RT} $$

In terms of mixed components, the following van der Waals mixing rule is used,

43 $$ a_{m} = \sum\limits_{i} {\sum\limits_{j} {x_{i} x_{j} a_{ij} } } $$

44 $$ b_{m} = \sum\limits_{i} {x_{i} b_{i} } $$

45 $$ c_{m} = \sum\limits_{i} {x_{i} c_{i} } $$

where, $a_{ij}$ is expressed in terms of the binary interaction parameter $k_{ij}$ as,

46 $$ a_{ij} = \left( {a_{i} a_{j} } \right)^{1/2} \left( {1 - k_{ij} } \right) $$

The fugacity of component $i$ in a mixture is computed as,

47 $$ \begin{aligned}   RT{\text{ln}}\left( {\phi _{i} } \right) &  =  - RT{\text{ln}}\left( {Z - B} \right) + RT\left( {\frac{{b_{i} }}{{v - b_{m} }}} \right) - \frac{{\sum\nolimits_{j} {x_{j} a_{{ij}} } }}{d}{\text{ln}}\left( {\frac{{Q + d}}{{Q - d}}} \right) + \frac{{a_{m} \left( {b_{i}  + c_{i} } \right)}}{{2\left( {Q^{2}  - d^{2} } \right)}} \\     & \quad  + \frac{{a_{m} }}{{8d^{3} }}\left[ {c_{i} \left( {3b_{m}  + c_{m} } \right) + b_{i} \left( {3c_{m}  + b_{m} } \right)} \right] \times \left[ {{\text{ln}}\left( {\frac{{Q + d}}{{Q - d}}} \right) + \left( {\frac{{2Qd}}{{Q^{2}  - d^{2} }}} \right)} \right] \\  \end{aligned}  $$

where, $d$ is estimated from mixed parameter set of van der Waals rule as,

48 $$ d = \left( {b_{m} c_{m} + \frac{{\left( {b_{m} + c_{m} } \right)^{2} }}{2}} \right)^{ - 1/2} $$

Additionally, the parameter $Q$ in Eq. (47) is evaluated as,

49 $$ Q = v + \frac{{b_{m} + c_{m} }}{2} $$

## Simulation algorithm and parameter estimation

The hydrate phase model of van der Waals and Platteeuw^25^ is based on the equality of the chemical potential of water between the equilibrium phases. The intermolecular potential function of Kihara then describes the interaction between the water molecules forming the cavity and the encaged guest molecule. In this study, these potential parameters of the Kihara function are regressed to the experimental hydrate phase equilibrium data obtained from open literature. The experimental range of this adopted phase equilibrium data is presented in Table 2. The parameters thus obtained are carried over to the phase equilibrium predictions in the presence of salt. Here, the binary interaction parameter of the Pitzer activity coefficient model is regressed to the experimental data.

### Parameter estimation

Firstly, the solubility estimate of the refrigerant in water is limited by the availability of Henry law’s parameters. To estimate these parameters, refrigerants solubility data and their variation with temperature were used and the parameters identified are reported in Table 3.

**Table 3:** Estimated Henry’s law constants and root mean square error (RMSE) of solubility estimates with respect to their experimental data^52–54^.

Hydrate former	References	$H_{iw}^{0}$	$H_{iw}^{1}$	$H_{iw}^{2}$	$H_{iw}^{3}$	RMSE
R13	^52^	−542.97	66,272.00	17.65	0.49	0.0038
R14	^52^	−791.07	116,450.00	−5.20	1.16	0.0115
R22	^53^	−469.98	69,376.00	10.07	0.46	1.05
R23	^53^	−462.77	50,094.00	5.28	0.70	0.4407
R32	^54^	1131.90	180,160.00	30.28	−2.49	0.3977
R125a	^54^	−101.79	18,732.00	59.53	−0.73	0.995
R134a	^53^	35.4120	−25.32	−2.55	−0.23	9.98E−07
CH_4_	^34^	−181.786	9112.582	25.04	−0.00015	–
C_2_H_6_	^34^	−286.441	13,369.40	37.55	−0.00230	–
C_3_H_8_	^34^	−316.490	15,922.70	44.32	0.0000	–
CO_2_	^34^	−159.868	8742.426	21.67	−0.00110	–

At the model estimate, the chemical potential of water in the hydrate phase $\mu_{{\text{w}}}^{{\beta - {\text{H}}}}$ (Eq. 1) balances out the potential in the bulk aqueous phase $\mu_{{\text{w}}}^{{\beta - {\text{aq}}}}$ (Eq. 6) as,

50 $$ \mu_{{\text{w}}}^{{\beta - {\text{H}}}} = \mu_{{\text{w}}}^{{\beta - {\text{aq}}}} $$

Consequently, Kihara potential parameters of collision distance $\left( \sigma \right)$, potential well depth $\left( \varepsilon \right)$ and core radius $\left( a \right)$ are identified by minimizing objective function based on the average absolute relative deviation (AARD) of the model estimate $\left( {P^{\bmod } } \right)$ compared to the experimental values $\left( {P^{\exp } } \right)$. The objective function employed is,

51 $$ {\text{\% AARD (P)}} = \frac{1}{N} \, \sum\limits_{i = 1}^{N} {\frac{{\left| {P_{i}^{\exp } - P_{i}^{\bmod } } \right|}}{{P_{i}^{\exp } }}\; \times } \;100 $$

**Figure 1:**
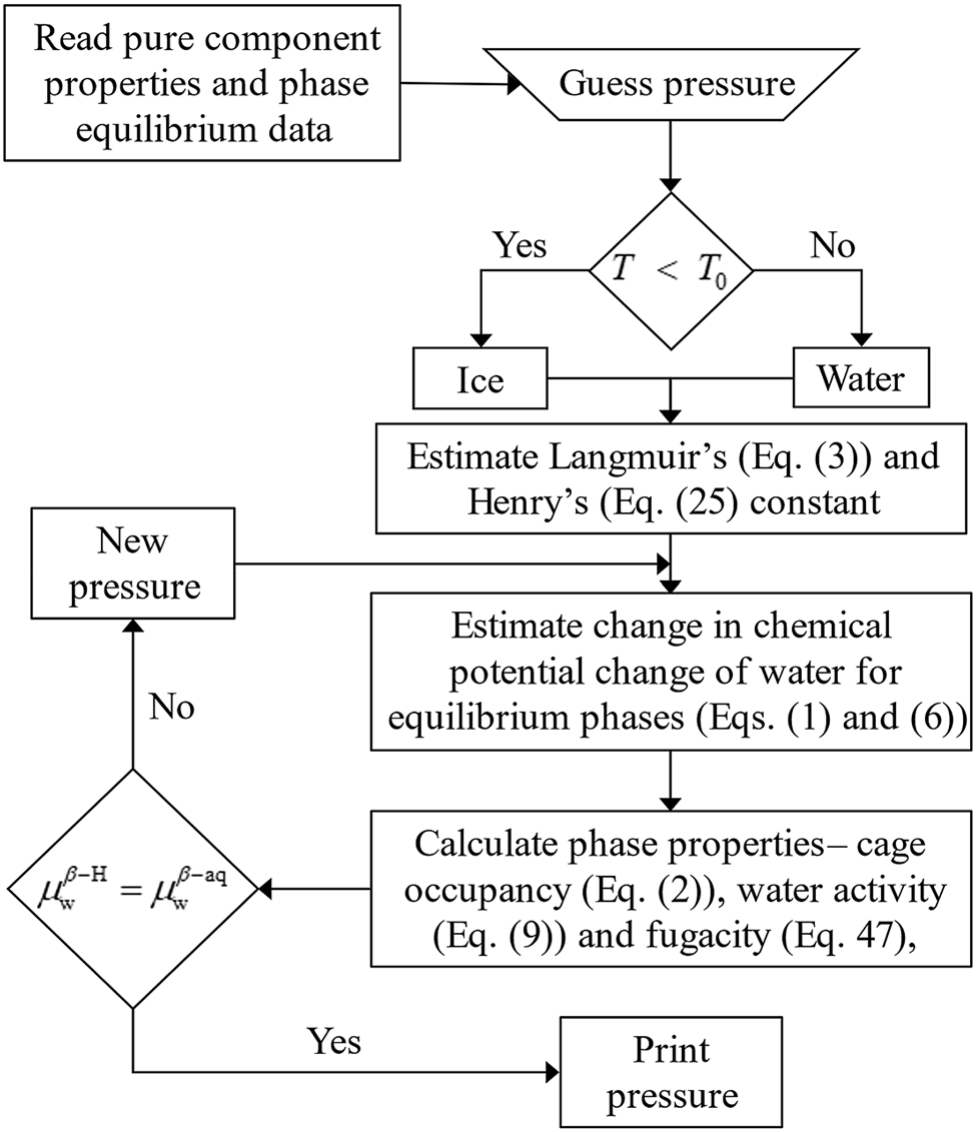
Computational algorithm to search hydrate equilibrium pressure.

To minimize this function, a simplex search algorithm is used from the MATLAB^®^ optimization toolbox, with a termination criterion set to 10^–5^. The pressure search algorithm to estimate the equilibrium pressure is presented in Fig. 1 and the obtained parameters are listed in Table 4.

**Table 4:** Kihara potential parameters regressed to experimental hydrate phase equilibrium data.

Guest	*a* (Å)	$\sigma$(Å)	$\varepsilon /k$(K)
R13	1.13160	2.7041	246.10
R14	0.72783	3.1356	153.89
R22	0.90019	2.8558	255.69
R23	0.9867	2.7990	188.90
R32	0.4053	3.2272	192.94
R125a	1.7867	2.6441	206.74
R134a	1.31135	2.7220	235.85
R152a	1.12910	2.8892	214.38
CH_4_	0.36783	3.1451	157.83
C_2_H_6_	0.30375	3.4744	172.69
C_3_H_8_	0.52233	3.7242	185.07
CO_2_	0.29943	3.4258	161.30

In the presence of electrolytes, the Pitzer activity coefficient model^37^ is used to describe their influence on the ideality of pure water. The interaction between the ions and refrigerant formers is then regressed to the experimental hydrate equilibrium data of refrigerants in electrolytic media. Table 5 lists these binary and ternary interaction parameters for different electrolyte and hydrate former pairs.

**Table 5:** Binary and ternary interaction parameters of Pitzer activity coefficient model.

Electrolyte	Hydrate former	$\lambda_{nc}$(cation–neutral)	$\lambda_{nc}$(anion–neutral	$\xi_{can}$(cation–anion–neutral)
NaCl	R13	−4.338	1.604	0.5474
	R22	−0.960	0.243	0.4725
	R23	−1.983	0.087	1.6320
	R134a	−2.647	0.084	0.5306
	R152a	0.1459	0.208	0.1950
	CH_4_	−5.884	0.648	0.7512
	C_2_H_6_	−0.3764	0.241	0.2609
	C_3_H_8_	−0.4729	0.234	0.3931
	CO_2_	−1.175	0.147	0.2262
MgCl_2_	R22	−1.120	0.487	0.2131
	R134a	−8.488	0.166	2.9654
KCl	R22	−3.303	0.243	0.1262
CaCl_2_	R134a	−0.056	0.166	−1.678

## Results and discussion

In addition to the equilibrium conditions, the thermodynamic model provides insight into the qualitative aspects of the equilibrium phases. To accurately predict these characteristics, we model the hydrate phase equilibrium data based on the statistical thermodynamic approach of van der Waals and Platteeuw^25^. The vapor and liquid phase non-idealities due to hydrate formers and salt ions are modelled using Patel Teja EoS^35^ and combined form of UNIQUAC^36^ and the Pitzer^37^ activity coefficient models, respectively. The estimates from the proposed model are compared with the existing works both in bulk pure and aqueous electrolytic phases. Model predictions are then used to estimate the hydrate phase characteristics. These attributes are further used to identify the best-suited refrigerant former for application to distributed load management and desalination systems.

### Hydrates in pure water

**Figure 2:**
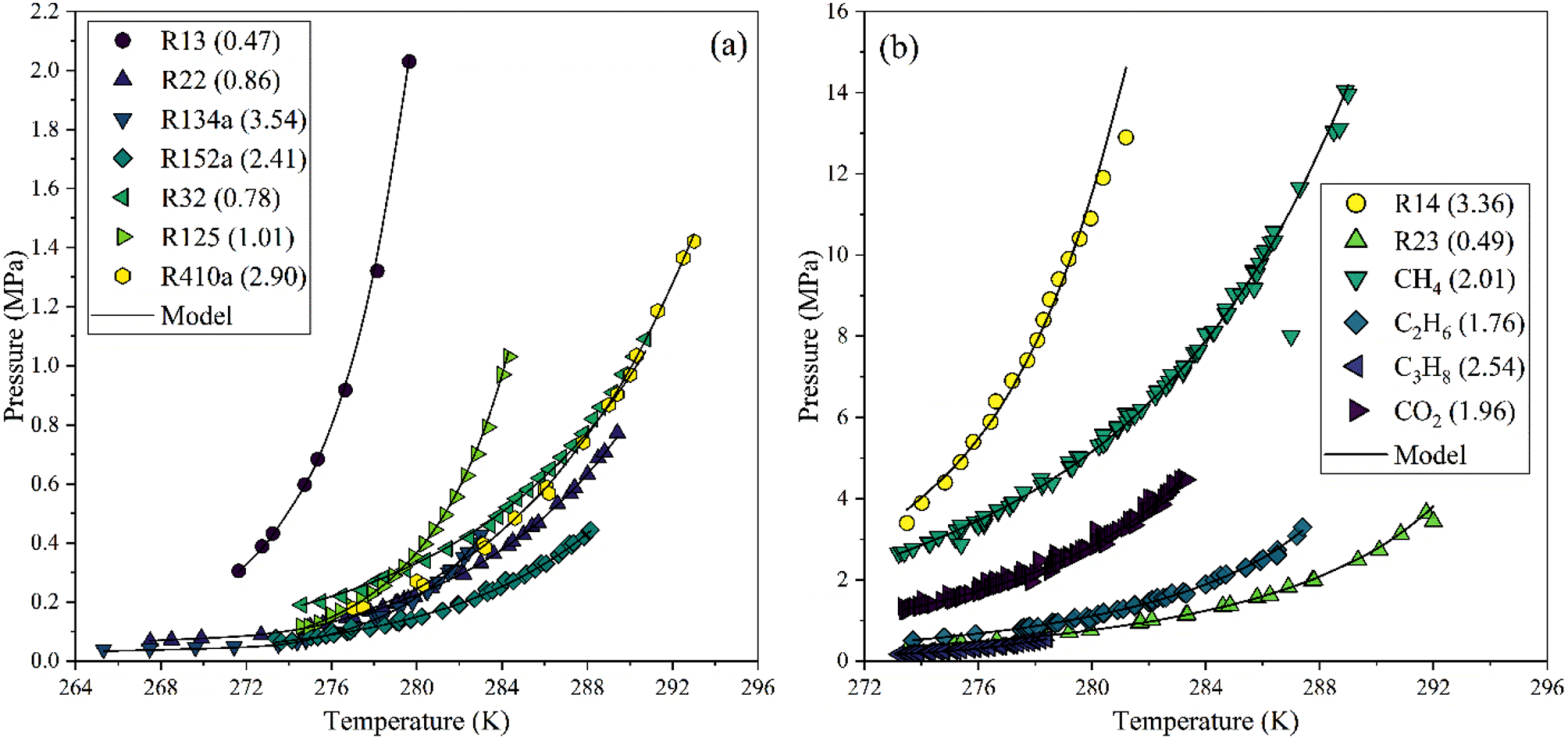
Model estimates of phase equilibrium conditions compared to the experimental data of chlorofluorocarbon (R13^40^), hydrochlorofluorocarbon (R22^42–45^), hydrofluorocarbon (R23^40–42,46^, R32^49,50^, R125^42,49,50^, R134a^42,47–50^, R152a^40,48^, R410a^42,47–50^), perfluorocarbon (R14^41^), and CH_4_^7^, C_2_H_6_^7^ and C_3_H_8_^7^ and CO_2_^7^ hydrates in pure water. The number in parenthesis against each legend quantifies the model %AARD in pressure.

The phase equilibrium estimates from the proposed model for hydrates of refrigerant formers in presence of pure aqueous phase are further discussed. Figure 2 presents the equilibrium conditions estimated using a pressure search algorithm for refrigerant hydrates in pure water. All the model estimates are in close approximation with their experimental datasets. The refrigerants being derivatives of small hydrocarbons provide much more lattice stabilizing interaction with the water molecules of hydrate lattice, resulting in milder operating conditions compared to their regular counterparts. Furthermore, from Fig. 2a, the fluorinated refrigerants are observed to provide more effective stabilization than the chlorine-based one. Interestingly, R410a, a mixture of R32 and R125a refrigerants, exhibits a higher quadruple point than either of them, suggesting a potent hydrate former for applications close to ambient temperature with much serviceable equilibrium pressure. The %AARD values exhibit close approximation to the experimental dataset having a minimum and maximum deviation of 0.47% and 3.54% for R13 and R134a refrigerants, respectively. Furthermore, the departure at a significantly higher pressure for R14 comes from the uncertainties and non-uniform trend of the experimental data, as shown in Fig. 2b. A comparison of the %AARD values from the model estimates with those reported in the literature is presented in Table 6. The comparison reveals overall improvements in the model estimate of hydrate phase equilibrium pressures across all refrigerant formers.

**Table 6:** A comparison of the %AARD-P values for hydrate equilibrium predictions for pure refrigerant hydrate formers.

Former	Number of data points (*N*)	T-range (K)	% AARD-P				
			Dongre and Jana^34^	Abolala et al.^30^	Karamoddin and Varaminian^29^	Ngema et al.^33^	This work
R13	8	273.15–281.15	–	0.59	–	–	0.47
R14	18	273.19–283.19	–	–	–	–	3.36
R22	32	267.50–289.40	–	0.89	3.20	–	0.86
R23	13	273.55–292.00	–	0.59	–	–	0.49
R32	32	274.60–290.13	–	0.91	–	–	0.78
R125a	23	274.60–284.30	–	0.77	–	–	1.01
R134a	24	265.31–283.13	–	3.8	–	5.30	3.54
R152a	40	273.39–282.15	–	–	3.20	–	2.41
R410a	18	277.00–293.00	–	3.98	–	0.79	2.90
CH_4_	83	273.20–289.00	2.48	–	–	–	2.01
C_2_H_6_	50	273.70–287.40	1.7	–	–	–	1.76
C_3_H_8_	50	273.20–278.40	2.65	–	–	–	2.54
CO_2_	165	273.36–283.30	1.98	–	–	–	1.96

To further test the extendibility of the proposed model to a wider types of hydrate formers, the hydrates of methane, ethane, propane and carbon dioxide are also studied. Table 4 reports the optimized Kihara pair potential parameters for these formers. The model estimates are observed to be in close aggrement with the experimental dataset. Besides, the deviations form the model are in line with those obtained by more complex approaches^34^. As evident from Fig. 2b, these hydrates are stably occurred in a very narrow ranges of temperature and pressure. This severely limits the applicability of such hydrates to DLMS as well as desaliantion, where the salt inherently acts as inhibitor to the formation of hydrates.

### Hydrates in electrolytic water

Above the eutectic point of salt, the solubility difference between the hydrate phase (micromolar concentrations) and the bulk aqueous phase (macromolar concentrations) inherently act as the driving force for salt rejection. This naturally facilitated movement of ions from the hydrate lattice to the unfrozen part of the system generates a gradient in salt concentration and temperature across the hydrate-water interface. As a hydrate layer solidifies, the sequential salt gradient onsets the formation of a new layer. However, microscopic instabilities at the hydrate–water interface lead to corrugations along the planer freezing front. Ions trapped in these corrugations then amount to the micromolar salt concentrations of the hydrate lattice.

**Figure 3:**
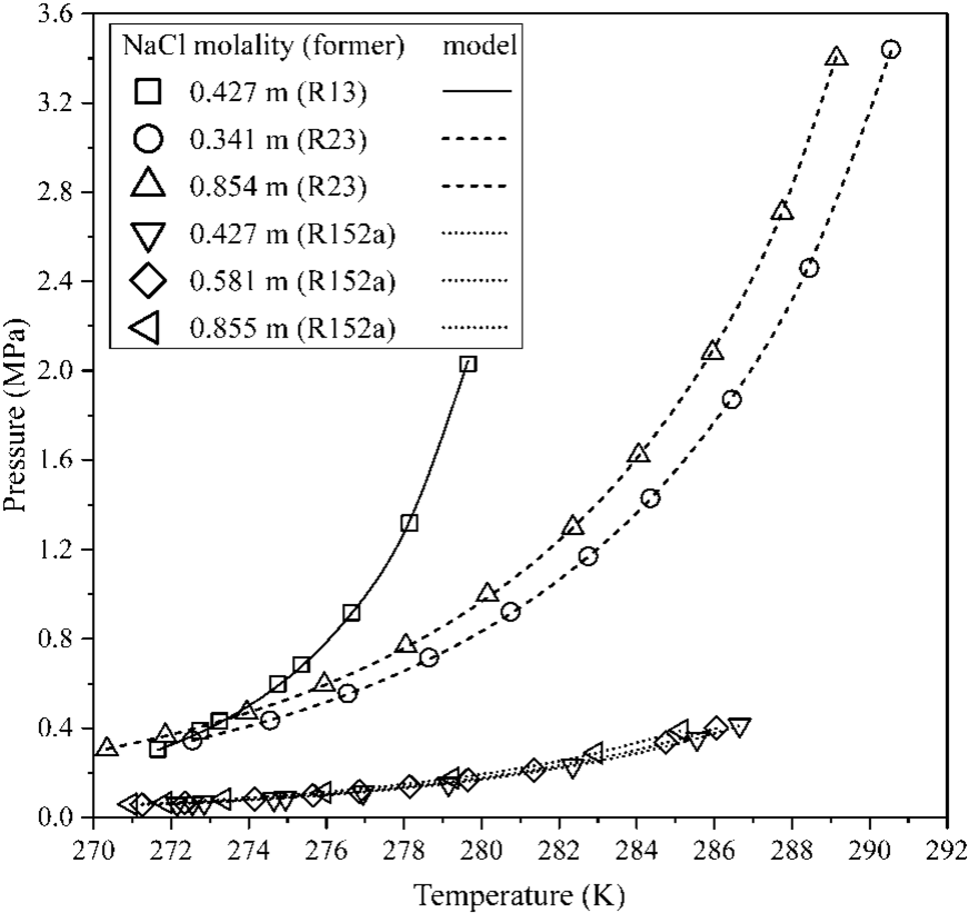
Experimental and estimated hydrate phase equilibrium conditions for hydrates of R13^40^, R23^40^ and R152a^40^ in presence of NaCl. The equilibrium conditions of R152a hydrates are marginally affected by varying concentrations of NaCl when compared to the other hydrate formers.

Such ionic interference results in an increase in the equilibrium pressures at the corresponding temperature of pure water hydrates. The proposed model uses the binary interaction parameters of the Pitzer activity coefficient model for regression to experimental data. The phase equilibrium model estimates for refrigerant hydrates of R13, R23, and R152a in electrolytic water are presented in Fig. 3. Here, NaCl concentration is observed to have a considerable influence on the equilibrium pressures of R13 hydrate compared to R23 hydrate. On the other hand, the hydrates of R152a have inconsequential changes to its equilibrium pressure with increasing NaCl concentration.

**Figure 4:**
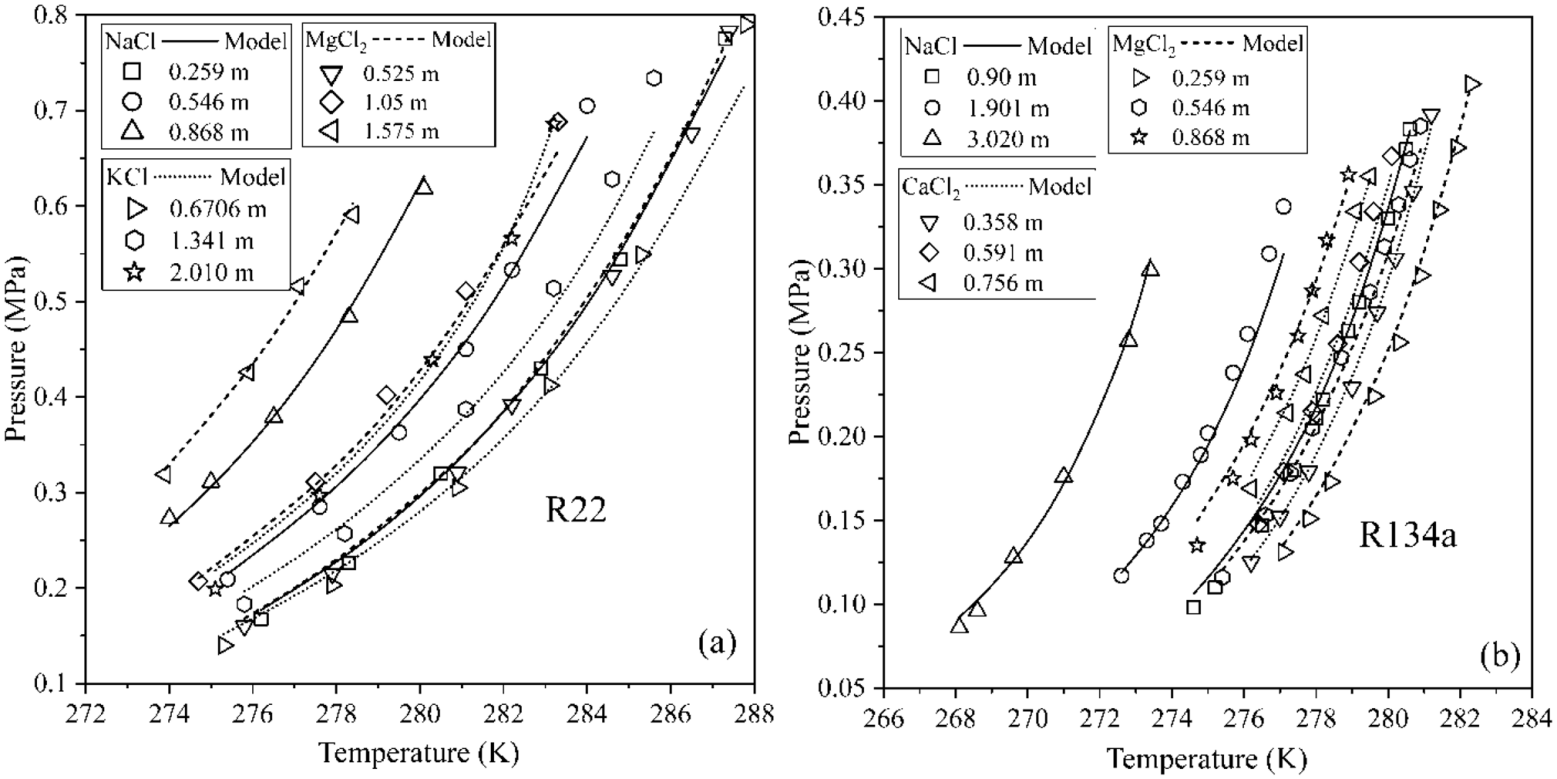
Comparison of phase equilibrium conditions of experimental data with model estimate for refrigerant hydrate formers at different salt concentration in water for (**a**) R22 and (**b**) R134a refrigerants. Model predictions in the presence of NaCl and MgCl_2_ salts are commonly depicted by solid and dashed lines, while the dotted lines portray KCl and CaCl_2_ salts for the hydrates of R22^45^ and R134a^31,33,51^ respectively. The individual molalities for each salt are grouped and presented correspondingly.

Figure 4 depicts the model performance of equilibrium pressure for R22 and R134a formers in the presence of different salts. The estimates obtained are in close agreement with the corresponding experimental datasets. On closer inspection, we can observe that, for one molar concentration of all salts, the inhibition effect is most predominant for CaCl_2_, followed by MgCl_2_ and NaCl. Furthermore, the aqueous electrolytic solution of KCl has the least inhibition effect amongst all these studied salts. Table 7 compares the sparsely available estimates of the literature, outperforming them all. The model estimates exhibit a maximum deviation of 7.94% for R22 refrigerant in aqueous KCl solution, while a minimum variation of 0.42% is observed for R13 refrigerant in NaCl aqueous solution. A considerable cavity distortion occurs at high pressures and salt concentrations, which alter the cage occupancies. These occupancy re-estimates require computationally expensive ab initio calculations^55^ or updates to the potential chemical estimate of the hydrate phase itself^56^. However, these modifications are neglected here, due to the only few model outliers.

**Table 7:** A comparison of the %AARD-P estimate for hydrate phase equilibrium for refrigerants formers in the presence of different salts.

Former	Salt	Molality (mol/kg)	Temperature range (K)	Number of data points (*N*)	% AARD-P			
					Dongre and Jana^34^	Ngema et al.^33^	Chun et al.^28^	This work
R13	NaCl	0.42	271.6–279.6	8	–	–	–	0.42
R22	NaCl	0.85–2.56	274.0–287.3	16	–	–	2.69	2.20
	KCl	0.67–2.01	275.1–287.8	17	–	–	4.14	7.94
	MgCl_2_	0.52–1.57	275.0–287.3	16	–	–	5.68	2.52
R23	NaCl	0.34–0.85	270.3–292.5	21	–	–	–	0.44
R134a	NaCl	0.09–3.02	268.1–280.6	27	–	5.3	–	4.69
	MgCl_2_	0.25–0.86	274.7–282.3	26	–	–	–	2.83
	CaCl_2_	0.43–0.89	276.2–281.2	21	–	–	–	2.19
R152a	NaCl	0.43–0.89	270.9–286.6	27	–	–	–	1.02
CH_4_	NaCl	0.52–5.40	267.8–299.1	97	7.38	–	–	9.65
C_2_H_6_	NaCl	0.52–4.27	265.4–284.7	24	3.62	–	–	6.85
C_3_H_8_	NaCl	0.43–4.27	265.2–276.6	54	–	–	–	9.42
CO_2_	NaCl	0.43–4.27	265.4–280.9	83	3.77	–	–	8.68

### Selection of refrigerant for hydrate applications

**Figure 5:**
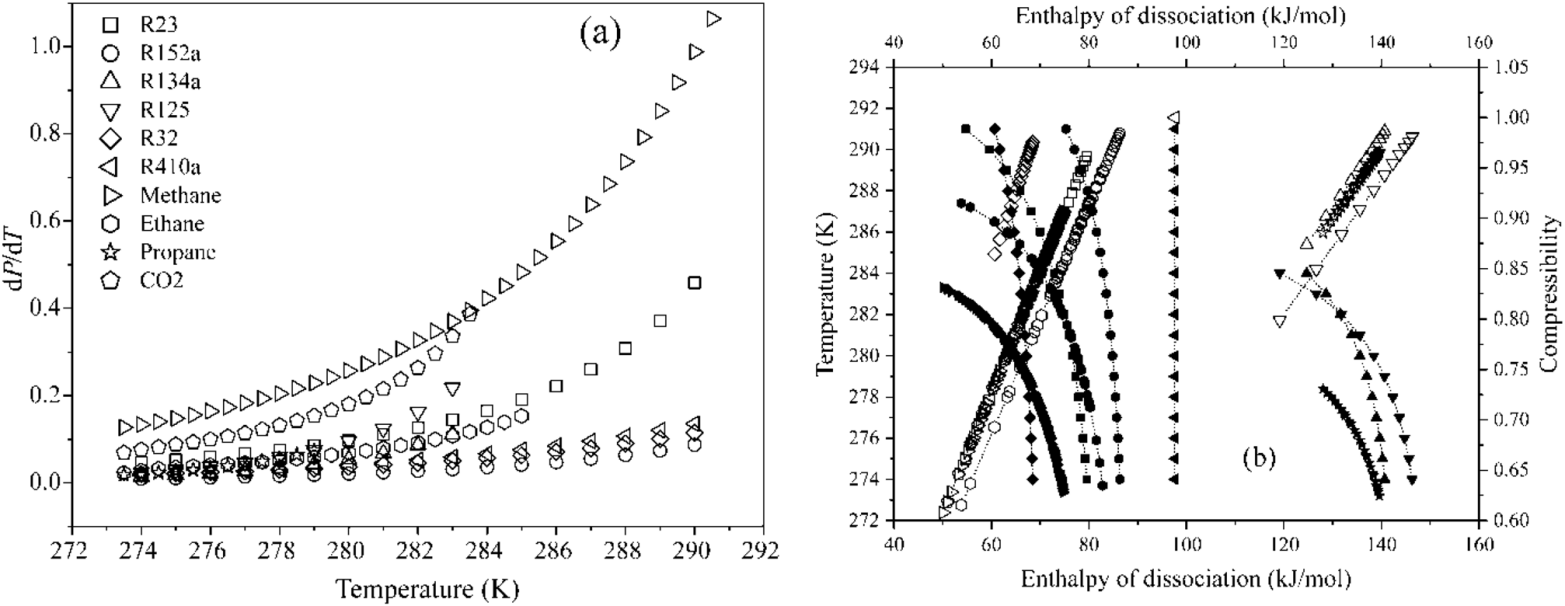
Model estimates of (**a**) change in the HLV equilibrium pressure per unit change in operating temperature (**b**) enthalpy of dissociation with respect to operating temperature and compressibility of refrigerant formers. The legends are shared between both figures and are detailed in figure (**a**). In figure (**b**), the solid and hollow interiors of legends denote the temperature and compressibility ranges, respectively.

The significant energy savings using the hydrate-based technologies can be achieved by selecting a former exhibiting milder formation pressure and appropriate heat density. Thus, refrigerant formers are distinguished based on model predictions quantifying the change in equilibrium pressure per unit change in operating temperature $\left( {{{{\text{d}}P} \mathord{\left/ {\vphantom {{{\text{d}}P} {{\text{d}}T}}} \right. \kern-\nulldelimiterspace} {{\text{d}}T}}} \right)$. Figure 5 plots this change, ${{{\text{d}}P} \mathord{\left/ {\vphantom {{{\text{d}}P} {{\text{d}}T}}} \right. \kern-\nulldelimiterspace} {{\text{d}}T}}$, and dissociation enthalpy of hydrates as a function of temperature and compressibility factor. The rapid fall in the enthalpy coupled with the narrow functional ranges of temperature severely limits the applicability of the hydrocarbon and CO_2_ hydrates. Further, we observe that R152a necessitates the least change in equilibrium pressure per unit change in temperature. Furthermore, hydrates of R134a and R32 both reach their quadruple point undergoing an equal change in equilibrium pressure. However, the R134a forms a much stable hydrate than R32 on account of its higher dissociation enthalpy. These enthalpies directly correlate the guest to cavity size ratio, interpreting the lattice cage occupancies. Thus, we can conclude that R125 hydrates exhibit a higher cage occupancy than the other refrigerant hydrates. Besides exhibiting a higher quadruple point than constituent formers, the dissociation enthalpy of R410a is insensitive to temperature and compressibility changes. Commonly, a decrease in the compressibility of the refrigerant formers increases the dissociation enthalpy of the refrigerant hydrates. The difference in the enthalpy of hydrate dissociation between different refrigerants at the same compressibility factor indicates its concentration dependence. These estimates are at phase equilibrium conditions and the actual operations would require a slightly higher pressure or lower temperature.

**Figure 6:**
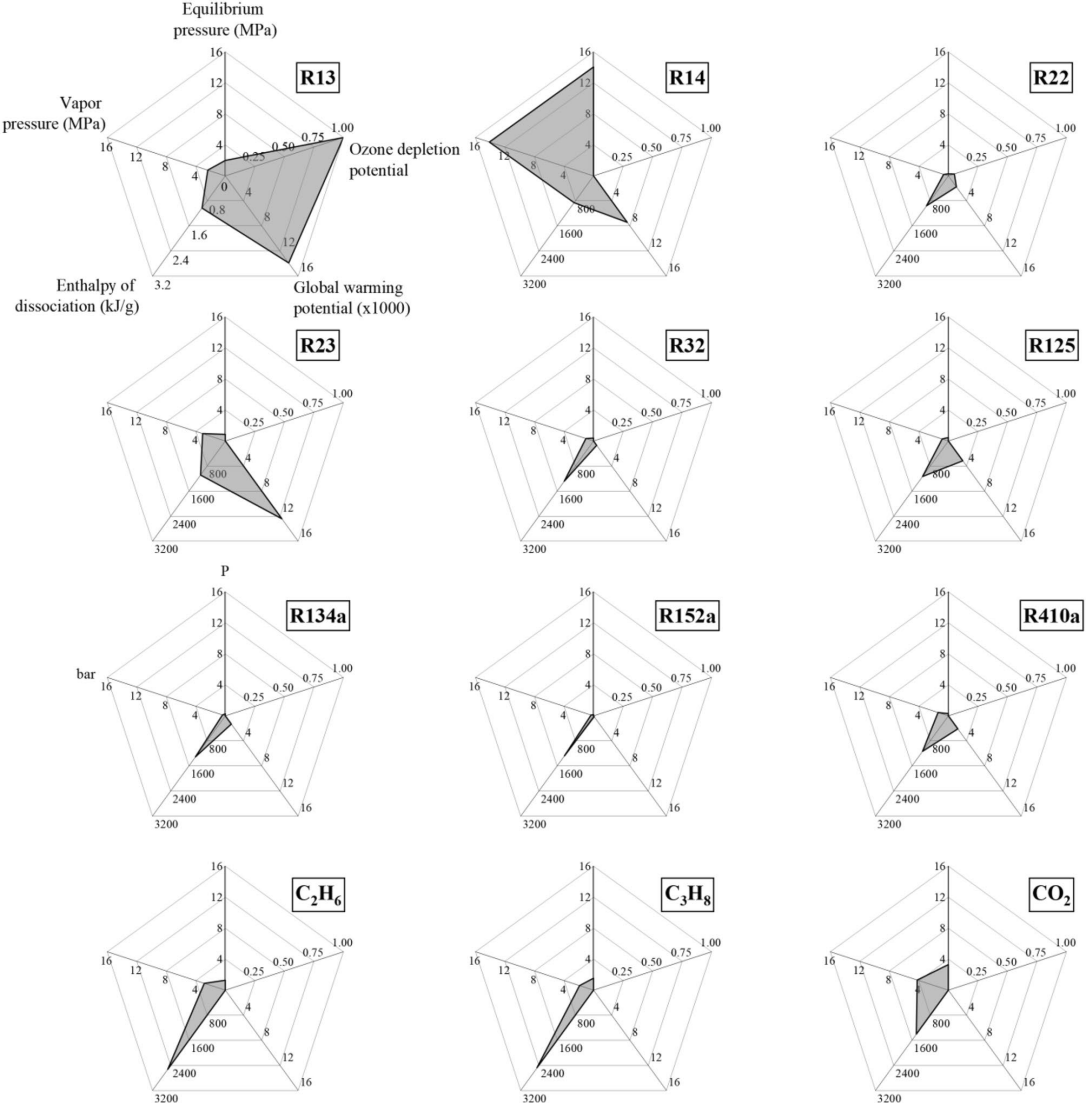
Comparison of the desirable properties of refrigerant formers for application to DLSM and cold storage applications. The annotations for all refrigerants are the same as given for R13. The R152a refrigerant hydrates exhibit most set of desirable properties for these applications.

A high heat storage density is most beneficial for the cold storage applications requiring PCMs. Since the average operating temperature of cold storage application is 278.15–285.15 K, a median temperature of 281 K is selected for identifying the suitable refrigerant former. Besides exhibiting low vapor pressure, a suitable former should be stable and have non-toxic chemical properties. Additionally, the refrigerant must meet the Montreal and Paris agreements possessing no global warming- (GWP) and ozone depletion- potential (ODP). Table 8 organizes the desirable properties with the predicted equilibrium pressures and the estimated dissociation enthalpies for hydrates of refrigerant formers in pure water at 281 K. A visual comparison of these properties between different refrigerants is depicted in Fig. 6 and reveals that apart from R14, all the other refrigerants have mild phase equilibrium pressures. Interestingly, between the structurally similar but functionally differing R13 and R14 refrigerant, chloride replacement with fluoride significantly impacts the equilibrium pressure. The other structurally comparable refrigerant formers like R22, R32, R125, and R410a exhibit a similar vapor pressure and thereby the associated equilibrium pressure and dissociation enthalpies. However, their associated GWP and ODP make them unsuitable for large scale operations. Finally, R134a and R152a exhibit the least phase equilibrium pressure and almost identical hydrate dissociation enthalpies (1300 kJ/kg) with no OPD. However, amongst the studied refrigerant formers, R152a, with an order of magnitude lesser GWP, emerges as the most suitable hydrate former for application to DLMS and cold storage.

**Table 8:** Refrigerant hydrate equilibrium and dissociation enthalpies estimated at 281 K.

Former	Predicted equilibrium pressure (MPa)	Enthalpy of dissociation (kJ/kg)	Ozone depletion potential^57^	Global warming potential^58^	Vapor pressure (MPa)
R13	2.01	1025.00	1.00	13,900.00	2.39
R14	14.05	854.79	0.00	7390.00	14.13
R22	0.26	956.10	0.05	1760.00	0.64
R23	0.86	1083.00	0.00	12,400.00	3.08
R32	0.37	1284.53	0.00	677.00	1.04
R125	0.46	1129.71	0.00	3170.00	0.85
R134a	0.27	1311.82	0.00	1300.00	0.39
R152a	0.17	1278.75	0.00	138.00	0.35
R410a	0.28	1134.57	0.00	2088.00	1.39
CH_4_	5.84	4374.03	0.00	25.00	12.27
C_2_H_6_	1.27	2530.18	0.00	5.5	2.86
C_3_H_8_	1.54	2477.77	0.00	0.02	1.9
CO_2_	3.29	1401.89	0.00	1	4.24

It is a fact that near ambient operating conditions (278–300 K) are often pursued in a quest to lower the operational and maintenance costs of seawater desalination. The unbiased natural exclusion of ions during the freezing of water can be exploited to achieve such desalination, albeit at low temperature. The clathrate of gas accommodates crystal formation at moderate temperatures, and it comes at the expense of elevated pressures. Further heightening these equilibrium pressures is the inhibitory nature of the saline environment. Thus, the necessity of a suitable hydrate former for seawater desalination is inevitable and it should function at mild pressure. Since the hydrates of refrigerant formers are stably found at mild operating conditions, they can aptly serve for water desalination.

**Figure 7:**
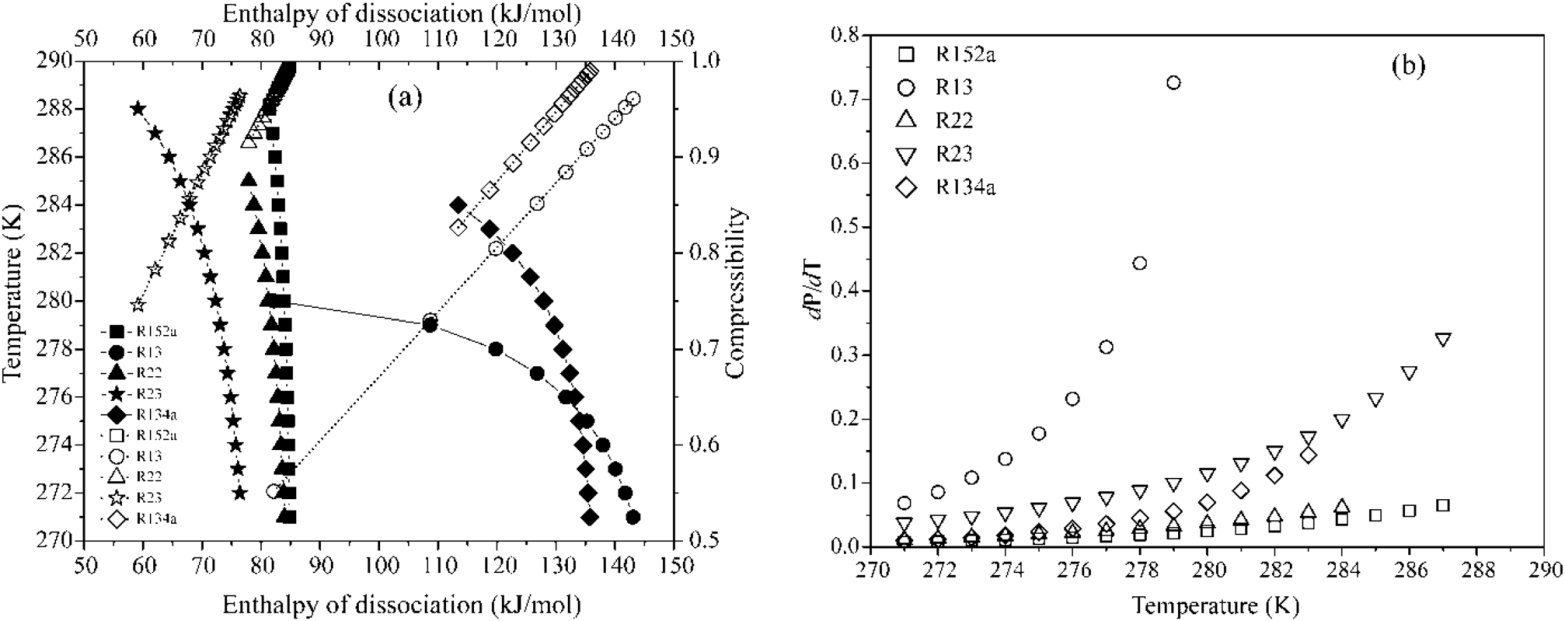
Model estimate of (**a**) enthalpy of dissociation as a function of operating temperature and compressibility of refrigerants, and (**b**) change in hydrate, liquid and vapor phase equilibrium pressure per unit change in the operating temperature in 3.5 wt.% NaCl solution. In figure (**b**), the solid legends depict the temperature range while the hollow legends indicate the compressibility range.

**Figure 8:**
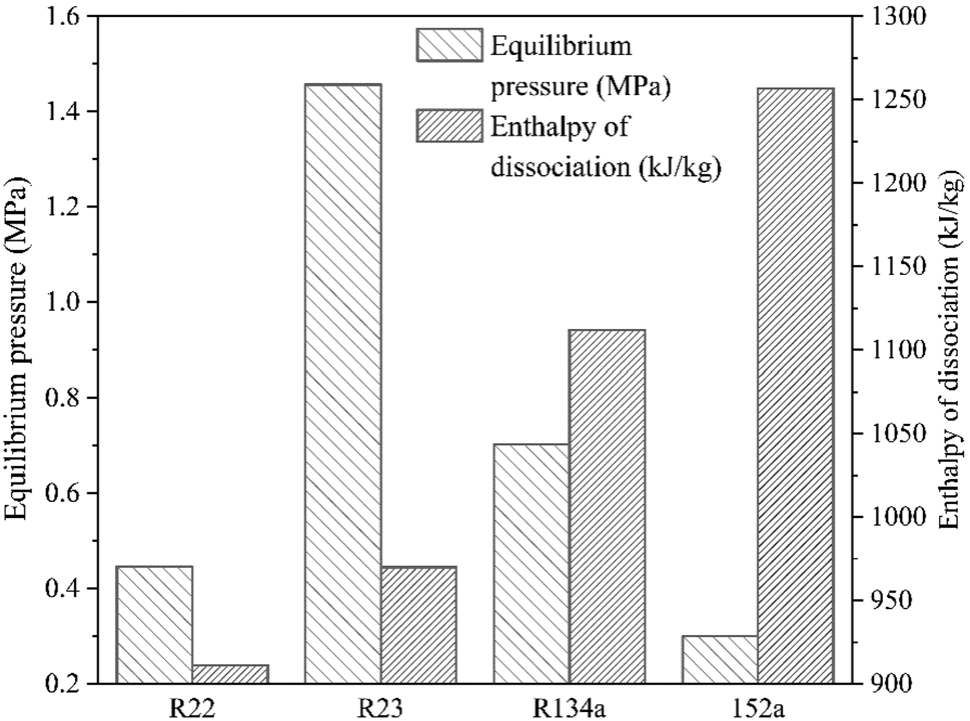
Comparison of predicted the per kilogram dissociation enthalpies and equilibrium pressure of refrigerant formers in 3.5 wt.% NaCl solution at 284 K.

The presence of salt increases this equilibrium pressure; however, it also lowers the dissociation enthalpies of hydrate crystals, needing utmost care during crystal handling. Figure 7 presents the model predictions for hydrate properties in the range of 273.15–288 K, while Fig. 8 compares the per kilogram dissociation enthalpies of refrigerant hydrates with their formation pressures at 284 K, in aqueous 3.5 wt.% NaCl solution. Here, the minimum equilibrium pressure and enthalpy of dissociation for the R22 hydrates make for most energy-efficient systems. However, the mild ODP and high GWP make it unsuitable for large scale deployment for HBD processes. The selection between R134a and R152 is a tossup because of the lower equilibrium pressure and higher dissociation enthalpies presented by the later former. Further making the selection difficult is the theoretically higher water to hydrate conversion of R134a than that of R152a, albeit at higher GWP.

## Conclusion

In this contribution, the quantitative and qualitative phase assessments provided by the developed thermodynamic models are proposed to identify the suitable refrigerant former for application to load management and desalination systems. Though numerous refrigerants are touted to be suitable formers for these applications, an apparent lack of consensus still exists. Since the phase equilibrium of the hydrate–liquid–vapor phases is often the first criterion for former selection, the statistical thermodynamic model of van der Waals and Platteeuw is used to model this associated experimental data. While Patel Teja equation of state describes the vapor phase, and UNIFAC and Pitzer activity coefficient models account for the non-idealities of the aqueous equilibrium phase. The percent average absolute relative deviation quantifies the model divergence from the experimental data, and a minimum of 0.47% and 0.42% is observed for R22 hydrates in pure and aqueous NaCl solution, respectively. Concurrently, model estimates for R134a and R22 exbibit maximum deviations in pure water and KCl-water solution, respectively. These model estimates accurately represent the experimental datasets and outperform the existing models under various phase equilibrium conditions. Further, CaCl_2_ exhibits the most inhibition effect towards hydrate formation, closely followed by MgCl_2_ and NaCl and KCl, showing the least repression. Model predictions at 281 K in pure water and 284 K in 3.5 wt.% NaCl solution are then obtained to mimic the bulk phase of concerned applications. Vapor pressure, compressibility and dissociation enthalpy predicted for these hydrates of refrigerant formers are compared. These properties along with the environmental friendliness of the R152a refrigerant makes its an expedient hydrate former for application to both DLMS/cold storage and desalination application. On the other hand, hydrates of R134a refrigerant offer hydrate properties close to R152a, albeit slightly environmentally unfriendly. The testing of model for systems involving methane, ethane, propane and carbon dioxide hydrates, in pure and aqueous NaCl solution further highlights the extendibility of the proposed approach. This thermodynamic framework towards identifying the application-specific refrigerant formers can thus help streamline the hydrate research for quickened commercial adoption of these hydrate-based technological solutions.

## Data Availability

The data that support the findings of this study are available from the corresponding author on reasonable request.
